# Supplementary material for: Content-rich biological network constructed by mining PubMed abstracts
Source: BMC Bioinformatics. 2004 Oct 8;5:147. doi: 10.1186/1471-2105-5-147 (PMC528731; doi:10.1186/1471-2105-5-147)
Supplement: Additional File 5 — The original Chilibot query results of the term "long-term potentiation (LTP)" and 22 other terms, limiting the latest references analyzed to the years 1990, 1995, 2000, and 2004. [file 1471-2105-5-147-S5.bz2 › chilibotAdditionalFile5/ltp2000/html/AMPA.html]

 


**AMPA** (Input: AMPA ) 

---


**Google Searches:** Entire Web  | EDU domain only  | PDF files only

.

**External Links:** OMIM |
LocusLink
| Swissprot
| GeneCards

  
**Maps of AMPA**

Simple
Complete
graph in
radiant
tree
square
layout.

**New Hypothesis !**

**Synonyms** 

- ampa   [PubMed]

**Synopsis**

- These results show that LTP and CaMKII activity drive **AMPA** Rs to synapses by a mechanism that requires the association between GluR1 and a PDZ domain protein.  Science, 2000 
  [25]

- In addition, our results strongly suggest that the mechanisms underlying STP of the **AMPA** receptor are postsynaptic in origin.  Learn MemLearn Mem, 2000 
  [23]

- The results suggest that calcium entering via NMDA receptors initiates the signalling cascade that results in enhanced **AMPA** currents because it is adding to cytoplasmic calcium from other sources to reach a threshold for this signalling pathway, or because calcium entering via NMDA receptors specifically activates this signalling pathway.  J Physiol, 1999 
  [22]

- In the present study we have examined the role of PKC in tetanus induced LTP of **AMPA** receptor mediated synaptic transmission in hippocampal slices obtained from adult rats.  Eur J Neurosci, 2000 
  [19]

- Because we have previously shown that acute ethanol can inhibit NMDA and **AMPA**,   Alcohol Clin Exp Res, 1999 
  [19]

- A study of polyphosphoinositide turnover showed that KA stimulated phospholipase C PLC activity was directly triggered by the **AMPA** KA receptor activity,  J Neurochem, 2000 
  [18]

- This reduction was in part caused by an apparently twofold reduction in mean quantal amplitude, as shown by recording **AMPA** receptor mediated miniature PSCs.  J Neurosci, 1999 
  [18]

- In particular, we discuss the role of both RS alpha amino 3 hydroxy 5 methyl 4 isoxazolepropionic acid **AMPA** and N methyl D aspartate NMDA glutamate receptors in the control of the short and long term efficacy of corticostriatal transmission.  Ann Neurol, 2000 
  [16]

- These experiments suggest that in clinical practice **AMPA** receptor blockade may have some advantage over N methyl D aspartate receptor antagonism, which is accompanied by severe memory impairment.  Brain Res Bull, 2000 
  [16]

- Finally, we will review data relevant to the issue of selective changes in **AMPA** binding after environmental enrichment and LTP.  Cell Mol Life Sci, 2000 
  [14]

- These results indicate that **AMPA** receptor phosphorylation is critical for synaptic plasticity,.  Nature, 2000 
  [14]

- Cyclothiazide CTZ, a potentiator of AMPAR, revealed slowly rising **AMPA** EPSCs at silent synapses.  Nat Neurosci, 2000 
  [12]

- The finding that modifications in synaptic strength are largely expressed by receptors of the **AMPA** subclass has focused attention on molecular mechanisms that affect their function and targeting.  Cell Mol Life Sci, 2000 
  [12]

- Induction of LTP or LTD altered the short term plasticity of transmission onto both pyramidal cells and interneuron CP **AMPA** synapses by a mechanism consistent with changes in release probability.  J Neurosci, 2000 
  [12]

- It is based on the assumption that, in diverse central nervous system structures, long term potentiation  [LTP]  depression LTP LTD of excitatory transmission depression potentiation of inhibitory transmission, LTDi LTPi is the result of an increasing decreasing the number of phosphorylated **AMPA** and NMDA GABA A receptors.  BiosystemsBiosystems, 2000 
  [11]
